# Communication channels to promote evidence-based practice: a survey of primary care clinicians to determine perceived effects

**DOI:** 10.1186/s12961-016-0134-z

**Published:** 2016-08-11

**Authors:** Ann Dadich, Hassan Hosseinzadeh

**Affiliations:** 1 School of Business, Western Sydney University, Locked Bag 1797, Penrith, NSW 2751 Australia; 2 Faculty of Medicine, University of New South Wales, Sydney, NSW 2052 Australia

**Keywords:** Knowledge translation, Sexual healthcare, Primary care, General practice, General practitioner, Practice nurse, Communication channels

## Abstract

**Background:**

Research suggests that the channels through which evidence-based practices are communicated to healthcare professionals can shape the ways they engage with, and use, this information. For instance, there is evidence to suggest that information should be communicated via sources that are deemed to be credible, like government departments, professional bodies and peers. This article examines the contention that information should be communicated via credible sources. More specifically, the article examines the different communication channels through which primary care clinicians learnt of resources on evidence-based sexual healthcare – namely, clinical aides and online training programs. Furthermore, the article determines whether these communication channels influenced the perceived impact of the resources.

**Methods:**

Primary care clinicians in Australia (n = 413), notably General Practitioners (n = 214) and Practice Nurses (n = 217), were surveyed on the *GP Project* – a suite of resources to promote evidence-based sexual healthcare within primary care. Survey items pertained to the source of information about the resources (or communication channel), perceived usefulness of the resources, frequency of use, subsequent contact with the Sexual Health Infoline and a sexual health clinic, as well as the perceived impact of the resources. To determine the relationships between the different communication channels and the perceived impact of the resources, a one-way ANOVA using Tukey’s post-hoc test, an independent sample *t*-test, a χ^2^ test, and a Kruskal–Wallis H test were performed where appropriate.

**Results:**

Of the respondents who were aware of the clinical aides (49.9%), the largest proportion became aware of these through an educational event or a colleague. Of those who were aware of the online training programs (36.9%), the largest proportion became aware of these through a professional body or government organisation, either directly or via their website. Although both resource types were reported to improve clinical practice, the reported use and the perceived impact of the resources were not influenced by the way the clinicians learnt of the resources.

**Conclusions:**

These findings cast doubt on the suggestion that the channels through which evidence-based practices are communicated to healthcare professionals shape the ways they engage with, and use, this information, as well as the perceived impact of this information. Given the importance of evidence-based practices, these curious findings suggest that the source of this information might be of little consequence.

## Background

In the epoch of neo-liberalism, which “*demands… greater ‘oversight’, ‘transparency’ and ‘accountability’*” [1], evidence-based practice is considered to be essential for quality patient care and (related to this) a viable health system [2]. It guides the allocation of limited resources and services, it informs government policy and funding priorities and, above all, it determines treatment options for the individual patient [3]. Evidence-based practice thus permeates the micro, meso and macro levels of the healthcare hierarchy [4].

Evidence-based practice largely reflects, “*reliance on research of programme effectiveness, explicit criteria to appraise evidence, and use of systematic reviews of intervention benefits and harms*” [5]. However, progressive understandings suggest it is much more than the mere overlay of research onto practice [6–8]. Evidence-based practice reflects a dynamic interplay between high-quality evidence from research, clinician expertise, patient (and potentially carer) preferences, available resources, and the context in which care is delivered – this may include the team a clinician collaborates with (within and beyond their organisation), leadership, organisational culture, the political climate, and local epidemiology, among others [9–11]. Given the interrelated and dynamic relationship between these (and potentially other) facets, evidence-based practice might be understood as care that is guided by high-quality evidence from research, but contextualised and appropriated to suit the situation at hand.

Despite the importance of evidence-based practice, clinicians do not consistently draw on the evidence available to them. Research reveals limited clinician adherence to indicators of appropriate care [12]. For instance, an Australian study found little adherence to evidence-based practices among clinicians across a range of health issues, and as such, suboptimal patient care:

“*Compliance with indicators of appropriate care at condition level ranged from 13%… for alcohol dependence to 90%… for coronary artery disease. For health care providers with more than 300 eligible encounters each, overall compliance ranged from 32% to 86%… Although there were pockets of excellence… the consistent delivery of appropriate care needs improvement*” [13].

The limited use of evidence-based practice can be costly on (at least) five levels, namely the individual, the social, the organisational, the economic, and the policy levels. At the individual level, patient recovery is likely to be hindered, if not ended prematurely [14]. At the social level, poor patient health is likely to contribute to the oft-cited burden of care among carers [15]; this in turn can strain (if not diminish) family ties and friendships [16].

At the organisational level, the limited use of evidence-based practice is likely to result in the misuse of limited public resources, including treatment, services and staff [17]. At the economic level are the associated financial costs. For instance, a national study on medications prescribed for people with obstructive airways disease estimated that, “*in 2008 prescribing of ICS* [inhaled corticosteroids] *outside treatment guidelines for asthma and COPD* [chronic obstructive pulmonary disease] c*ost the Australian Government at least $*[AUD] *2.7 million, with a further $*[AUD] *200,000 cost to patients*” [18]. Similarly, research in the United States concluded that diagnostic errors represent the “*most common, most costly and most dangerous of medical mistakes*”, with an inflation-adjusted, 25-year payment sum of $US 38.8 billion and mean claim of $US 386,849 [19].

At the policy level, the limited use of evidence-based practice suggests misspent time and effort on the development of protocols and dissemination strategies that have limited influence on clinician behaviour [20]. This is particularly because the development of some guidelines is estimated to cost approximately $CAD 100,000–150,000 [21]. This is not to suggest that protocols – like clinical practice guidelines – are not important, particularly given their role in determining appropriate care as well as benchmarking efforts; but rather, it might be naïve to simply assume that superimposing didactic codes of behaviour onto various and complex health services will consistently change clinical practices.

Many factors impede evidence-based practice, including limited awareness of, and familiarity with, these practices, as well limited confidence in the information [22–24]. For instance, in a study involving psychiatrists, nurses and dental hygienists, Asadoorian et al. [25] found that, “*All the psychiatrists had some level of mistrust of research publications and the ‘evidence’*” [25]. More recently, Lenzer [26] has described “*Why we can’t trust clinical guidelines*”, citing conflicts of interests among guideline panellists, which compromise guideline development (perceived or otherwise). This might partly explain the use of credible figures and organisations to promote evidence-based practice.

In accordance with social influence theory, the promotion of evidence-based practices is said to be aided by respected individuals and organisations with beliefs, assumptions and norms that are comparable to the targeted clinical group, or to which the target group aspires [27, 28]. For instance, in a study involving a nationally representative sample of 1212 primary care physicians, Han et al. [29] found that clinical guidelines developed by a national professional body for specialists or a national not-for-profit organisation were deemed most influential, relative to those developed by a government taskforce or national professional bodies for physicians. This might suggest that physician perceptions of the guidelines might have been shaped (in part) by their perceptions of the organisation responsible for developing them. Conversely, others have reported clinician concern about the involvement of industry in guideline development [30, 31]. It thus appears that, as clinicians seek affiliation with their profession and their professional peers, their behaviours and views are likely to be shaped by the individuals and organisations they hold in high-regard and/or are guided by. This is aptly demonstrated by research on the potential value of opinion leaders [32, 33]. As Moulding et al. [34] explain:

“*The perceived opinions of peers and opinion leaders play a major part in influencing the attitudes of individual practitioners and, most importantly, their decisions to act on new information… Other groups with a stake in guidelines include policy makers, researchers, the press, and the healthcare industry… and their influence should be taken into account when planning guideline development and implementation*.

*Hence, social influence based strategies for implementing guidelines might include… group education, the use of opinion leaders, and mass media education strategies such as publication in journals or campaigns*” [34].

Theoretically, research on social influence suggests several reasons to account for this effect. For instance, contemporary understandings of French and Raven’s [35] seminal typology of social power bases refer to soft and hard bases of power [36–38]. Soft bases that have particular relevance to this article include positive expert power, where an individual is influenced by the perceived expertise of another; positive referent power, where an individual endeavours to align their behaviours with those they identify with; direct informational power, where an individual is influenced by those who present information perceived to be logical; and personal reward power, where an individual is influenced by the, “*promise of monetary or nonmonetary compensation*” [39]. A hard base of power relevant to this article is formal legitimate power, where an individual is influenced by the weight of another’s recognised position.

Similarly, of the seven core principles of social influence identified by Cialdini [40], four are particularly relevant to the focus of this article. First, the principle of instant influency/primitive automaticity suggests that, when prompt decisions are required (as is often the case among clinicians), individuals may be inclined to revert to a “*single-piece-of-good-evidence*” [40] to expedite what might otherwise be a complex decision-making process. Second, social proof implies there might be a perception of safety-in-numbers, whereby a behaviour is deemed to be proper when others are performing it (perceived or otherwise). Third, liking proposes that individuals are inclined to be influenced by those they are familiar with, and/or are fond of – this might be consequent to regular contact and/or perceived similarity [41]. Finally, the principle of authority suggests that individuals are likely to be influenced by those who assume authoritative positions, particularly because of the knowledge, wisdom and power they are assumed to hold – as such, guidance from authoritative figures might help to shortcut complex decision-making processes.

Given the potential value of social influence theory, this article investigates its role in promoting evidence-based healthcare. More specifically, the article examines the different communication channels through which primary care clinicians learnt of resources on evidence-based sexual healthcare, namely, clinical aides and online training programs. Furthermore, the article determines whether these communication channels influenced the perceived impact of the resources.

Sexual healthcare within the primary care sector constitutes an appropriate context for three key reasons. First, despite the prevalence of sexually transmissible infections (STIs) [42–45], the provision of sexual healthcare is limited, particularly within primary care [46, 47]. For instance, a cluster randomised controlled trial on chlamydia screening in general practice found that limited time, limited clinician understanding of associated benefits, and clinician concern about broaching sexual health with patients hindered clinician capacity to deliver evidence-based sexual healthcare [48]. This can have serious implications as some STIs remain asymptomatic and have long-term effects if left untreated [49, 50].

Second, within a number of Western nations, the primary care sector is experiencing significant reform [51, 52]. In Australia, for instance, current developments aim to “*shift the centre of gravity of the health system from hospitals to primary health care*” [53]. This is supported by (1) the nationwide introduction of Medical Locals, at the time of this study [54] – “*primary health care organisations established to coordinate primary health care delivery and tackle local health care needs and service gaps*” [55]; (2) considerable investment in the primary care workforce; (3) the establishment of GP Super Clinics across Australia; and (4) increases to after-hours patient access to primary care. As such, identifying ways to promote evidence-based primary care represents an area worthy of academic attention.

Third, primary care clinicians are being called to alleviate the strain on public sexual health clinics [56]. As stated in a government sexual health strategy, “*The size of some priority population groups is such that a strategic objective for specialist clinics and Area-based sexual health programs must be to work with general practice to reduce barriers to access*” [57]. These three reasons lend sexual healthcare in the Australian primary care sector as an appropriate context for this study. Before presenting this research, the following section describes the aforementioned clinical aides and online training programs, which form part of the *GP Project*.

### GP Project

The New South Wales STI Programs Unit (NSW STIPU) developed nine resources, all of which were guided by clinical guidelines [58], to improve evidence-based sexual healthcare within general practice in the Australian state of NSW. Given the focus of this article, findings pertaining to only four are reported – namely, two clinical aides and two online training programs. This is because they represent two modes of communication and were promoted and disseminated to primary care clinicians via comparable channels.

**Fig. 1:**
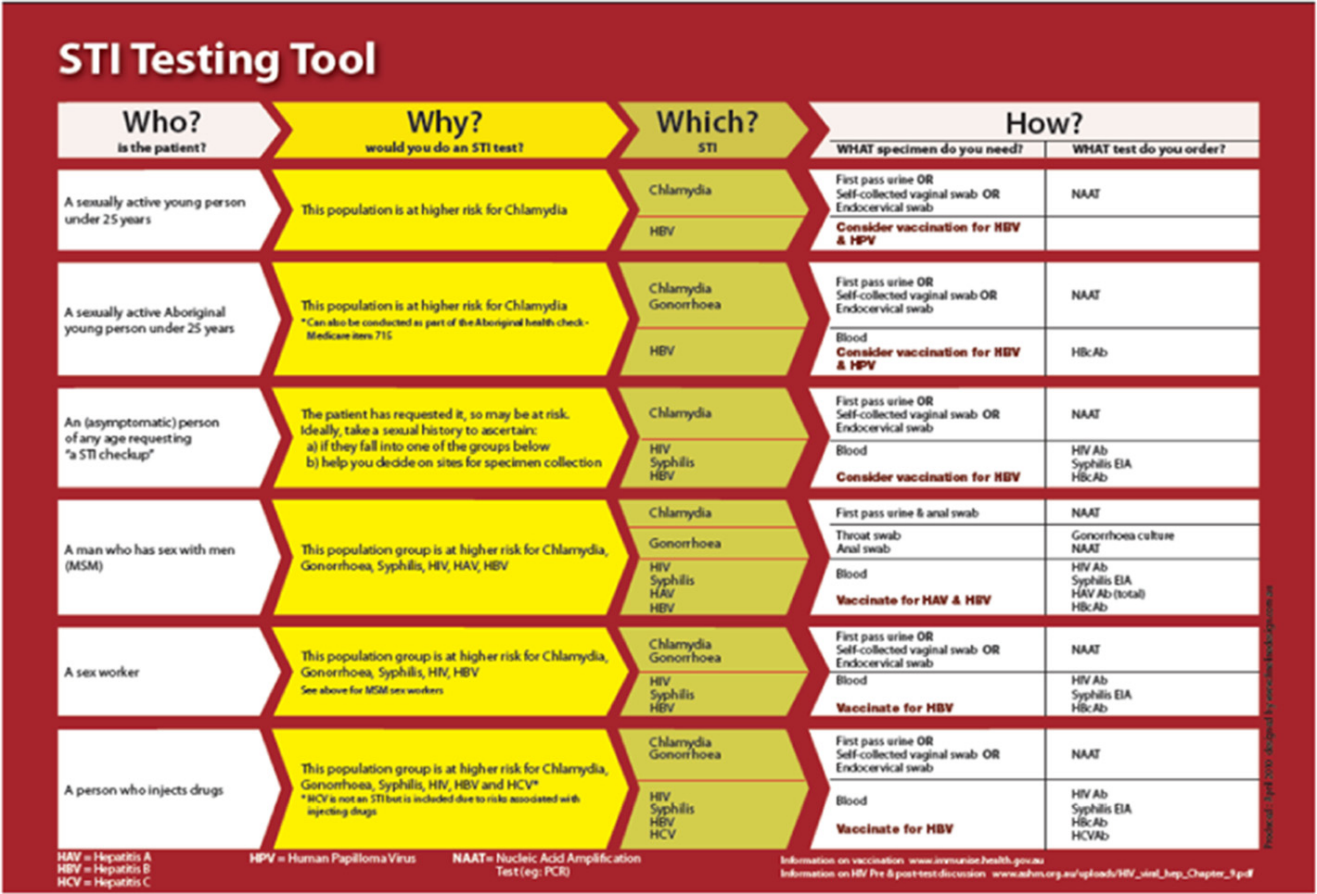
STI Testing Tool

The clinical aides include the STI Testing Tool and the Practice Nurse Postcard, which were designed for GPs and Practice Nurses, respectively. The STI Testing Tool guides sexual health consultations, with reference to the identification of at-risk patients, appropriate screening tests and the specimens required, appropriate ways to initiate and manage a sexual health consultation, a guide to documenting a brief sexual history, appropriate ways to broach contact tracing with patients, as well as referral information (Fig. 1).

**Fig. 2:**
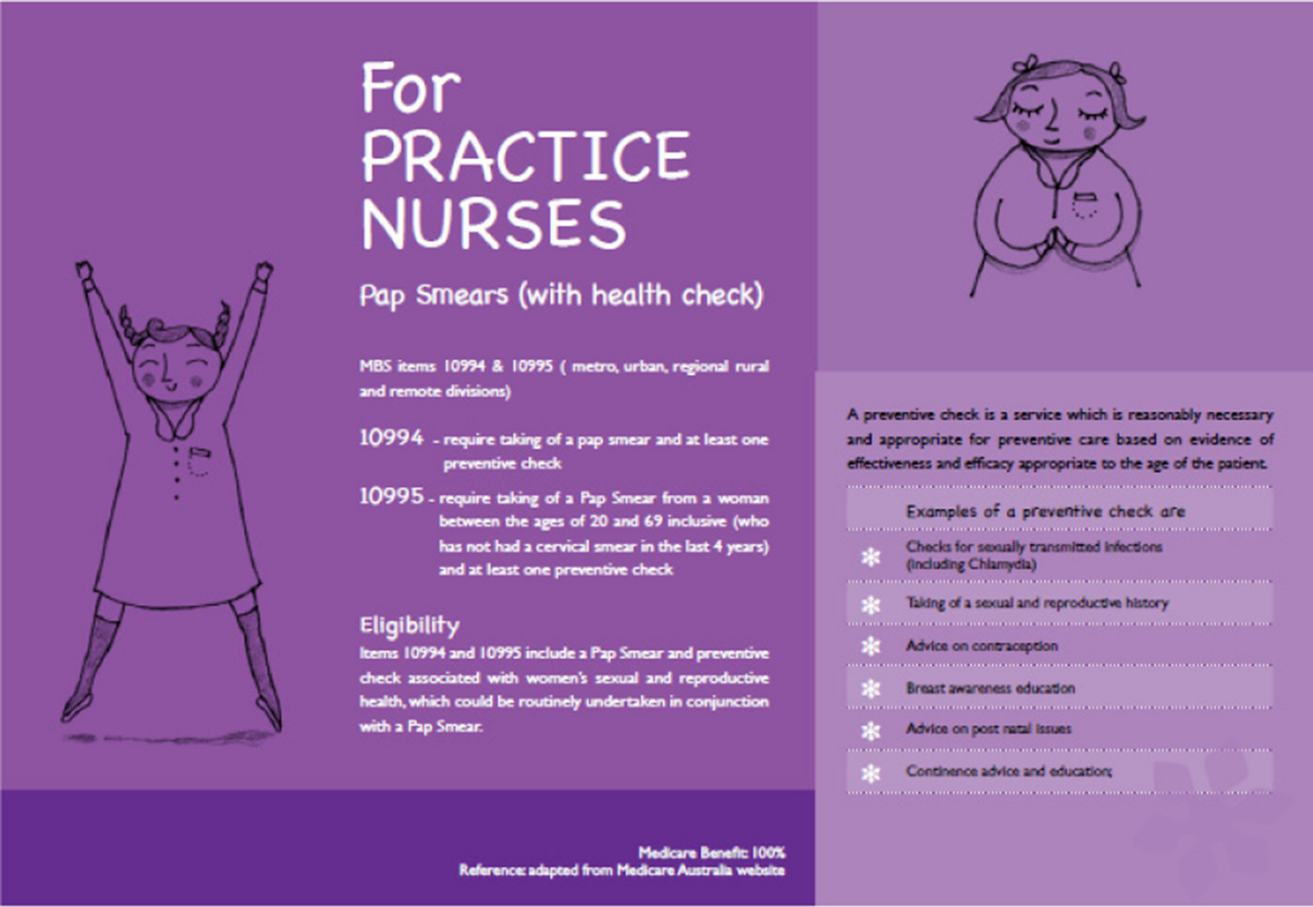
Practice Nurse Postcard

The Practice Nurse Postcard was devised to enable Practice Nurses to undertake a preventative women’s health check, including a pap smear. It provides information on the health check, prompts to document a brief sexual history, information to support the management of chlamydia, as well as contact details for further resources (Fig. 2).

Following their development, the two clinical aides were primarily promoted and disseminated to GPs and Practice Nurses via Divisions of General Practice, which (at the time of study) were professional bodies that supported primary care clinicians and promoted general practice. The aides were also promoted at educational events attended by primary care clinicians, other professional bodies, like the Australian Practice Nurses Association (APNA), as well as websites of professional bodies and government organisations, like NSW STIPU.

**Fig. 3:**
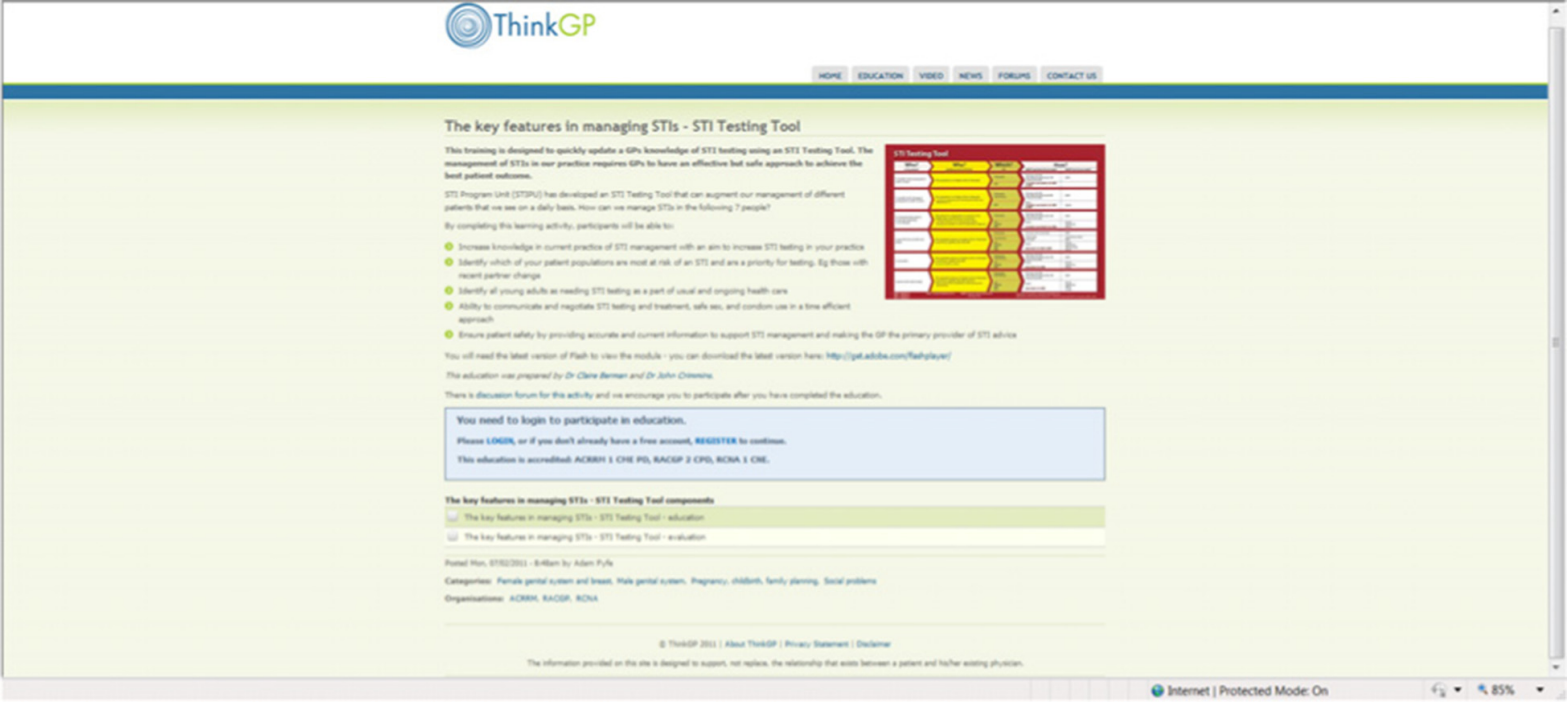
Online STI Testing Tool GP Training

The two online training programs include the Online STI Testing Tool GP Training and the Online STI Practice Nurse Training, which were designed for GPs and Practice Nurses, respectively. Developed and delivered by ThinkGP, a provider of online education to healthcare providers [59], the Online STI Testing Tool GP Training takes approximately 60 minutes to complete and includes seven clinical cases, offering participants the opportunity to apply their skills and knowledge (Fig. 3). These abilities are tested through the completion of questions after each clinical case.

**Fig. 4:**
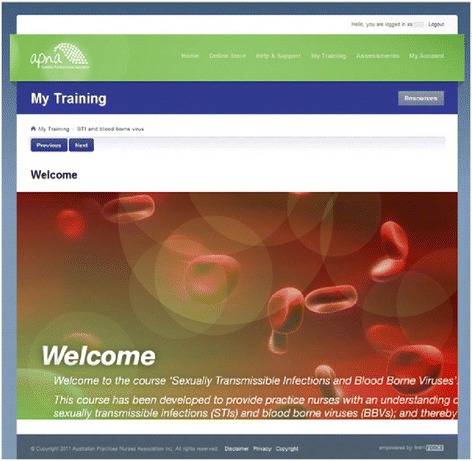
Online STI Practice Nurse Training

As part of the APNA Online Training program [60], the Online STI Practice Nurse Training focuses on understanding and managing STIs, blood borne viruses, HIV, as well as viral hepatitis (Fig. 4). The program takes approximately 90 minutes to complete. At the end of each section, participants are presented with a summary to reinforce key lessons. Upon completion, participant abilities are assessed.

Subsequent to their development, the two online training programs were primarily promoted to GPs and Practice Nurses via the websites of professional bodies and government organisations. More specifically, the Online STI Testing Tool GP Training was advertised on the ThinkGP and NSW STIPU websites, while the Online STI Practice Nurse Training was promoted via the APNA website.

Following the promotion and dissemination of these four resources, this study was conducted to determine the associated effects of the different communication channels. More specifically, this article questions how the primary care clinicians learnt of these resources and what were the perceived effects, particularly on the delivery of evidence-based sexual healthcare.

## Methods

Following clearance from the appropriate ethics committee (approval number: H8886), primary care clinicians – notably, GPs and Practice Nurses – who were practicing in NSW, were recruited with the assistance of relevant professional bodies, including 33 Divisions of General Practice, their auspicing body, as well as an independent provider of online education. These organisations included project information in their communications to GPs and Practice Nurses, which included email, facsimiles, website postings, and newsletters. Respondents were offered hard copies of clinical guidelines for their participation. In the absence of membership data from the Divisions of General Practice, or the mailing lists of the auspicing body and the independent provider of online education, it is not possible to estimate the population size from which the sample was recruited. However, current information suggests there are approximately 8585 GPs and 30,777 nurses collectively affiliated with all Primary Health Networks within the state of NSW, which have since replaced the Divisions of General Practice following government reforms [61, 62].

Over the course of 5 months (August 2011 to January 2012), the primary care clinicians were invited to complete an anonymous and a confidential online survey comprised of closed and open-ended items. The purpose of the survey was to evaluate all nine resources that collectively formed the GP Project, details for which are published elsewhere (WITHHELD FOR BLIND REVIEW) [63–66]; only items relevant to this article are reported. In addition to demographic information, one survey item queried the source of information about the resources – that is, the communication channel (‘How did you become aware of the [resource]?’); one item pertained to the perceived usefulness of the resources (‘How useful is the [resource]?’); one item queried the frequency of use (e.g. ‘How frequently do you use the [resource]?’, ‘How frequently do you use information from the [resource]?’); two items gauged subsequent contact with the Sexual Health Infoline (a government-funded resource on sexual healthcare) and a sexual health clinic (e.g. ‘Since using the [resource], have you contacted the NSW Sexual Health Infoline?’, ‘Since completing the [resource], have you contacted a sexual health clinic about a patient?’); and three items determined the perceived impact of the resources (e.g. ‘After using the [resource], my ability to raise the topic of STIs with my patients has improved’). Respondent views on the perceived impact of the clinical aides and the online training were measured via three items using a 5-point Likert scale, where 1 denoted ‘Strongly Disagree’ and 5 ‘Strongly Agree’, whereas 0 denoted ‘Unsure’. The internal consistency of the three-item measures was acceptable to excellent; the Cronbach’s alpha coefficient for the clinical aides was 0.73, and 0.93 for the online training.

Univariate descriptive statistics were calculated to determine respondent demographic information and their views on the perceived impact of the resources. Following this, a one-way ANOVA using Tukey’s post-hoc test, an independent sample *t*-test, a χ^2^ test, and a Kruskal–Wallis H test were performed where appropriate to determine the association between respondent demographics and key variables, namely, communication channel, where an educational event denotes direct informational power (a soft base of power) and a colleague represents positive referent power (a soft base of power), while professional bodies and government organisations epitomise formal legitimate power (a hard base of power), frequency of use and perceived impact.

## Results

### Respondents

A total of 413 primary care clinicians participated in this study, including 214 GPs and 217 Practice Nurses. Most respondents were female (74.3%) and had graduated in Australia (72.3%; Table 1). The highest proportion was between 36 and 55 years of age (63.6%). On average, the GPs had 15.4 years of GP experience, while the Practice Nurses had 7 years of experience as a Practice Nurse. More than a quarter of the respondents consulted patients in a language other than English (27.7%). The highest proportions worked with up to three GPs (38.7%) and Practice Nurses (69.9%) within their primary practice. The highest proportion of respondents indicated that fewer than 10% of their patients were from culturally and linguistically diverse backgrounds (43.4%); however, most reported that 10–50% of their patients were under 25 years of age (68.5%). As such, many of these clinicians consulted patients deemed to be at-risk of STIs, as per clinical guidelines [58].

**Table 1:** Clinician demographic information (n = 413)

Demographic Information	Frequency	%
Profession		
GP	214	49.7
Practice Nurse	217	50.3
Sex		
Male	108	25.7
Female	313	74.3
Age, years		
<36	76	17.6
36–55	274	63.6
>55	81	18.8
Country of Graduation		
Australia	305	72.3
Overseas	117	27.7
Consultation Language		
English only	317	74.1
English and a non-English language	111	25.9
GPs at Primary Practice		
1–3	161	38.7
4–6	136	32.7
>7	119	28.6
Practice Nurses at Primary Practice		
0	45	11.0
1–3	286	69.9
≥4	78	19.1
CALD Patients		
<10%	185	43.4
10–50%	149	35.0
>50%	72	17.0
Unsure	20	4.6
Patients <25 years		
<10%	81	19.0
10–50%	293	68.5
>50%	32	7.5
Unsure	22	5.0

CALD, culturally and linguistically diverse

Given the limited availability of demographic data on the profile of primary care clinicians in NSW [67], it is difficult to ascertain whether the respondents were representative of this cohort. However, data on the sex and age of GPs and all registered nurses in this state suggest they were not entirely representative of this cohort. This is because NSW GPs are mostly male (63.1%) and approximately one-third are over 55 years of age (31.6%). Similarly, although NSW registered nurses are mostly female (89.6%) (akin to the respondents), approximately one-fifth are over 55 years of age (21.2%), which differs from the respondents. These differences might be due to the population-based recruitment approach (as opposed to purposive sampling) and/or the voluntary nature of participation. Nevertheless, the respondents represented diverse geographical locations (as indicated by Division affiliation) and supported diverse patient populations, including patients from culturally and linguistically diverse backgrounds and young patients.

### Clinical aides

Approximately half of all respondents indicated that they were aware of the STI Testing Tool or the Practice Nurse Postcard (49.9%; Table 2). This interesting finding suggests that, despite considerable effort to inform GPs and Practice Nurses of these resources via their Divisions of General Practice, educational events, professional bodies and relevant websites, the impact of this effort was limited. The largest proportion of respondents reported to have become aware of these clinical aides through an educational event or a colleague (40.1%) or their Division of General Practice (33.5%). Most of the respondents reported to have used the clinical aides (68.7%); of these, more than half found them extremely useful or very useful (57.9%). More than one-third of those who reported to have used the aides indicated that they always or often used them during their practice (36.5%).

**Table 2:** Awareness, use and perceived impact of the clinical aides and online training (n = 413)

	Clinical Aides		Online Training	
	Frequency	%	Frequency	%
Awareness				
Aware of resource				
Yes	215	49.9	152	36.9
No	216	50.1	260	63.1
Source of information re the resource				
GP Division	56	33.5	29	23.2
Educational event or a colleague	67	40.1	24	19.2
NSW STIPU or a professional body	25	15.0	35	28.0
Website	19	11.4	37	29.6
Use				
Resource used				
Yes	138	68.7	30	21.9
No	63	31.3	107	78.1
Perceived usefulness				
Extremely/very useful	80	57.9	19	65.5
Useful	55	39.9	9	31.0
Not very/at all useful	3	2.2	0	0.0
Unsure	0	0.0	1	3.5
Frequency of use				
Always/often	50	36.5	12	41.5
Sometimes	47	34.3	10	34.5
Occasionally/never	37	27.0	6	20.6
Unsure	3	2.2	1	3.4
Impact				
Assists with clinical practice				
Strongly agree/agree	106	78.0	24	82.8
Neutral	24	17.6	4	13.8
Strongly disagree/disagree	6	4.4	0	0.0
Unsure	0	0.0	1	3.4
Improved GP-ability to raise the topic of STIs with patients or improved Practice Nurse-ability to take a brief sexual history				
Strongly agree/agree	95	69.8	23	85.2
Neutral	36	26.5	3	11.1
Strongly disagree/disagree	5	3.7	0	0.0
Unsure	0	0.0	1	3.7
Improved GP-ability to order appropriate STI tests or improvedPractice Nurse ability to identify at-risk patients or test for chlamydia				
Strongly agree/agree	103	76.3	23	82.2
Neutral	27	20.0	3	10.7
Strongly disagree/disagree	5	3.7	0	0.0
Unsure	0	0.0	2	7.1
Subsequent contact with the Sexual Health InfoLine				
Yes	24	17.6	4	13.8
No	94	69.1	23	79.3
Unsure	18	13.2	0	0.0
Subsequent contact with a sexual health clinic				
Yes	44	31.9	10	34.5
No	79	57.2	17	58.6
Unsure	15	10.9	0	0.0

Most of the respondents who reported to have used the aides indicated that they helped to improve their clinical practice (78.0%). For instance, most of those who used the aides reported improvements in their patient consultation skills (69.8%) – more specifically, the GPs reported they were better able to broach the topic of STIs with patients, while the Practice Nurses reported they were better able to take a brief sexual history. Similarly, most of those who reported to have used the aides reported superior clinical skills (76.3%) – while the GPs reported they were better able to order appropriate STI tests, the Practice Nurses reported they were better able to test patients for chlamydia. Furthermore, after using the clinical aides, close to one-third of the respondents indicated they had contacted a sexual health clinic in relation to a patient (31.9%).

To determine whether particular respondents were drawn to particular communication channels, the relationships between demographic information – notably, sex, age and profession – and the different sources of information about the clinical aides were examined. Following Kruskal–Wallis H analyses, no significant relationships were found (sex: χ^2^ (1, N = 431) = 2.00, *P* = 0.15; age: χ^2^ (2, N = 431) = 3.41, *P* = 0.18; profession: χ^2^ (1, N = 431) = 0.50, *P* = 0.47). As such, particular respondents were not attracted to particular sources of information, like their Division of General Practice, educational events or colleagues, NSW STIPU or professional bodies, or websites.

To determine whether particular communication channels influenced the reported use of the clinical aides, a χ^2^ analysis was conducted. No significant relationship was found (χ^2^ (3, N = 431) = 1.77, *P* = 0.62). As such, particular sources of information did not appear to influence frequency of use.

To determine whether particular communication channels influenced the perceived impact of the clinical aides, one-way ANOVA using Tukey’s post-hoc test were conducted. No significant relationships were found (*F* (3, 116) = 0.33, *P* = 0.79). For instance, no significant mean difference in the perceived impact of the aides was detected between those who learnt about them via their Division of General Practice (M = 11.53, SD = 1.46) and those who learnt about them via a website (M = 11.54, SD = 1.66). As such, particular sources of information did not appear to influence the perceived impact of the clinical aides.

### Online training

Over one-third of all respondents indicated they were aware of the online training programs (36.9%; Table 2). This notable finding demonstrates the limited reach of the aforesaid websites to inform GPs and Practice Nurses of these programs. Those who were aware of the programs chiefly learnt of these via a website (29.6%), NSW STIPU or a professional body (28.0%), or their Division of General Practice (23.2%). Of those who were aware of the training, approximately one-fifth reported to have completed it (21.9%). Of these, over two-thirds found the training extremely useful or very useful (65.5%) and more than 40% indicated that they always or often used the information they learned during their practice (41.5%).

Most of the respondents who completed the online training programs advised that the content aided their clinical practice (82.8%). For instance, most reported improvements in their patient consultation skills (85.2%) – more specifically, the GPs noted they were better able to raise the topic of STIs with patients, while the Practice Nurses noted they were better able to take a brief sexual history. Similarly, most of those who completed the training reported superior clinical skills (82.2%) – while the GPs reported they were better able to order appropriate STI tests, the Practice Nurses reported they were better able to identify patients at-risk of STIs. Furthermore, after completing the training, over one-third of the respondents indicated that they had contacted a sexual health clinic in relation to a patient (34.5%).

To determine whether particular respondents were drawn to particular communication channels, the relationships between demographic information – notably, sex, age and profession – and the different sources of information about the online training programs were examined. Following Kruskal–Wallis H analyses, no significant relationships were found (sex: χ^2^ (1, N = 431) = 5.38, *P* = 0.06; age: χ^2^ (1, N = 431) = 2.90, *P* = 0.23; profession: χ^2^ (1, N = 431) = 1.89, *P* = 0.16). These findings suggest that particular respondents were not attracted to particular sources of information, like their Division of General Practice, educational events or colleagues, NSW STIPU or professional bodies, or websites.

To determine whether particular communication channels influenced the reported use of information sourced from the online training programs, a χ^2^ analysis was conducted. No significant relationship was found (χ^2^ (3, N = 431) = 1.23, *P* = 0.74). As such, particular sources of information did not appear to influence frequency of use.

To determine whether particular communication channels influenced the perceived impact of the online training programs, one-way ANOVA using Tukey’s post-hoc tests were conducted. No significant relationships were found. For instance, no significant mean difference in the perceived impact of the training was detected between those who learnt about them via educational events or colleagues (M = 12.14, SD = 1.77) and those who learnt about them via a NSW STIPU or a professional body (M = 12.50, SD = 1.06). As such, particular sources of information did not appear to influence the perceived impact of the online training programs.

## Discussion

This article examined how primary care clinicians learnt about two resources on evidence-based sexual healthcare – namely, clinical aides and online training programs – and the associated perceived effects, particularly on clinician practices. Guided by contemporary understandings of French and Raven’s [35] research, findings suggest that the respondents were largely drawn to elements of both soft and hard bases of power [36–38]. More specifically, of those who were aware of the clinical aides, the largest proportion learnt of these resources through an educational event, which might arguably represent a source of information perceived to be logical, thus demonstrating direct informational power; or a colleague, that is, a peer they identify with, thus demonstrating positive referent power [39]. Of those who were aware of the online training programs, the largest proportion learnt of these resources through a professional body or government organisation, either directly or via their website. As sources of authoritative information, professional bodies and government organisations may represent formal legitimate power. Although particular respondents were not attracted to particular sources of information, these findings collectively demonstrate the potential sway of soft bases of power (notably, direct informational power and positive referent power), as well as a hard base of power (notably, formal legitimate power) among primary care clinicians sourcing information on evidence-based sexual healthcare.

Despite this notable finding, the results suggest these three forms of power had no significant influence on the reported use of the resources or clinician practices. Although most of the respondents who used the resources cited improved clinical practices, the channel through which they learnt of these resources did not influence frequency of use or perceived impact on clinical practices. These findings reveal limitations in the potential sway of the channels that represent direct informational power, positive referent power, and formal legitimate power. More specifically, they suggest that, while educational events, colleagues, professional bodies and government organisations might help to inform clinicians about information on evidence-based practices, they are unlikely to influence how frequently clinicians report using the resources or the perceived effects. These findings appear to challenge extant literature, which suggests clinician behaviours are likely to be shaped by those they hold in high regard and/or who they are guided by [32–34].

In light of social influence theory [27, 28] and contemporary understandings of social power [36–38], the limited influence of these communication channels on resource-use and perceived effects represents a curious find. This is largely because individuals and organisations that are respected, liked, and/or assume authoritative positions are said to shape behaviour [40]. This is especially the case for those perceived to have direct informational power, positive referent power, or formal legitimate power. Yet, the results from this research reveal limits to their sway. An explanation for this curious find is beyond the scope of this study; however, the finding substantiates a need for further study to determine the boundaries of these (and other) elements of social power among primary care clinicians.

Despite the value of the findings from this study, four limitations warrant consideration. First, as noted, the respondents do not constitute a representative sample of NSW GPs or Practice Nurses [67, 68]. Second, the survey did not solicit respondent attitudes towards, or perceptions of the different channels through which they learnt about the resources. Third, the cross-sectional design might have influenced the perceived impact of the resources; this is largely because of the reliance on recall. Fourth, given the reliance on self-reports, respondent perceptions could not be verified.

## Conclusions

The findings from this study are important for four key reasons. First, given the demonstrated limited reach of mechanisms like professional bodies, educational events and relevant websites, the findings underscore the need for further research to identify effective and efficient approaches to inform clinicians of available resources. This includes a consideration of how information communicated by an individual, rather than an organisation, shapes clinician perceptions and subsequent use of the information, the preferred ways to communicate this information, and whether different evidence-based practices require different communication channels – for instance, should the communication of evidence-based sexual healthcare differ from that for evidence-based palliative care, and if so, how? Such empirical research will optimise the effective and efficient use of the limited resources at the disposal of organisations responsible for health promotion. Second, they suggest that individuals and organisations perceived to hold direct informational power, positive referent power, and/or formal legitimate power might serve as effective communication channels to inform clinicians of resources on evidence-based practices. Third, given that these individuals and organisations might have limited bearing on the frequency of resource-use or the perceived impact on clinical practice, other sources of influence (and the elements of power they represent) warrant consideration. Fourth, these findings provide a platform for future research on the limits of social power (perceived or otherwise), and the factors that help and hinder the influence of individuals and organisations on clinicians.
